# Dental Policy Lab 1 - towards a cavity-free future

**DOI:** 10.1038/s41415-021-3723-3

**Published:** 2021-12-17

**Authors:** Christopher R. Vernazza, Nigel B. Pitts, Catherine Mayne, Marco E. Mazevet

**Affiliations:** 41415112851001 School of Dental Sciences, Newcastle University, Newcastle upon Tyne, UK; Newcastle upon Tyne Hospital NHS Foundation Trust, Newcastle upon Tyne, UK; 41415112851002 Faculty of Dentistry, Oral & Craniofacial Sciences, King´s College London, Tower Wing, Guy´s Hospital, London, SE1 9RT, UK

## Abstract

Although many dental professionals argue that prevention of oral diseases, including dental caries, will benefit both the patient and public finances, a paradigm shift has yet to happen in most countries. The literature has demonstrated that caries prevention and control is possible, but authorities have yet to implement health systems that allow patients to stay in a good health state. 'Policy Labs' are an innovative policy-making initiative that allow a positive collaboration between the many stakeholders around a given policy issue. In July 2017, 24 international experts, including representatives of both international and European Chief Dental Officers associations, were gathered for the first Alliance for a Cavity-Free Future/King's College London Dental Policy Lab to identify the main barriers for a change, and concrete actions to facilitate a policy shift towards increased resource allocation in prevention. A comprehensive report and well-received infographic summarising the key recommendations (explored in this paper) were produced to explain the situation and highlight the value of a cavity-free world to policymakers, demonstrating where change is needed. The first Dental Policy Lab proved to be an efficient way to generate new ideas and concrete ways to implement them, and has led to several subsequent initiatives worldwide.

## Key points

- Progress within both dental practice and health systems is needed to enable a paradigm shift towards preventive approaches to dentistry.
- 'Policy Labs' are an innovative policy-making initiative that allow a positive collaboration between the many stakeholders around a given policy issue.
- The creation of effective dental payment systems, equipping and expanding the dental workforce, shifting public and industry behaviour, and demonstrating the value of a cavity-free world are essential steps in working towards this shift.

## Introduction

Despite professionals, public health organisations and dental associations widely advocating for a greater focus on prevention, limited progress has occurred in reorienting dental health systems including general dental practice services. Dental caries is still the most prevalent condition worldwide in adults and affects more than 600 million children worldwide.^1^ This situation, even in developed countries such as the UK, has been described as 'criminal and unacceptable',^2^ with caries remaining the most common reason for hospital admissions in children, with over 25,000 admissions of 5-9-year-olds in England in 2018-2019.^3^

Though the prevalence of dental caries has been decreasing in many countries, there is an increasingly uneven distribution of caries across populations, following a distinct socioeconomic gradient, with an increase in caries prevalence in the ageing population, who are keeping their teeth for longer than before.

### The use of preventive approaches in oral health (including dental caries)

There is widespread acceptance that it is possible to maintain teeth in a healthy state and keep teeth from going down the 'repeat restorative spiral', which is costly in both health and financial terms. Many strategies have been described in the literature, ranging from community-based interventions such as water fluoridation to chairside dietary advice and minimally invasive dentistry.^4^ Although terminology differs across various specialist silos, prevention is usually classified into three categories (Table 1).

**Table 1:** Three stages of prevention applied to dental caries

Stage	Application to dental caries
Primary prevention	Prevention of disease in the absence of disease carried out to variable extents by separate public health groups (such as community-based fluoride strategies as a foundation for oral health)
Secondary prevention	Prompt detection of early-stage disease in order to provide effective arrest and/or regression prior to the cavity stage
Tertiary prevention	For more advanced (cavitated) stages of lesion severity, this aims to prevent further hard tissue destruction while restoring function and aesthetics and preventing the initiation of new disease. However, restorative care is often provided when not yet needed according to contemporary guidance (tooth structure destroying invasive surgical care provided, but often without any control of the aetiological or risk factors to prevent recurrence of caries)

Minimally invasive dentistry, risk management, and caries management and control techniques have been well described in the literature and are now a reality for general dental practice.^4^^,^^5^^,^^6^^,^^7^

Some general dental practitioners (GDPs) and their teams have been trained in minimally invasive dentistry (now increasingly referred to as minimum intervention oral healthcare [MIOC]) through their undergraduate curricula or continuing professional development (CPD). However, these techniques are often not rewarded in dental contracts and are unknown to patients, thus giving little incentive to perform them.

### Shifting the focus to prevention

Changing the focus of professionals, patients and systems to prevention is a complex task, involving many stakeholders and requiring significant change in expectations and behaviour as well as the potential re-design of health systems.^4^^,^^8^^,^^9^^,^^10^ There is an increasing recognition that implementing changes such as these requires policy change and that achieving policy change often hinges on the economic aspects of any such suggested change. These policy changes (in parallel to upstream changes to secure integrated primary prevention) rely on shifting resources to prevention at a system and an individual level. Despite a large body of evidence on which preventive interventions work at a clinical level, there is much less evidence about the economic implications of preventive clinical interventions or policy changes.

One of the tools required to facilitate a shift in resource allocation is an appropriate payment system for dental teams. Modern dental health systems (whether they are publicly or privately funded) have been built around fee-for-service payments which provide a financial incentive to treat diseases that have already appeared.^11^ There is very little remuneration for keeping patients in a healthy state, and primary and secondary preventive care have traditionally been left out of dental contracts. Health system managers and policymakers may be reluctant to move away from treatment activity-based payments due to a desire to ensure dental professionals are using public funds to maximum effect and the difficulties with measuring activity based around prevention.

## Materials and methods

### Policy Labs

Policy Labs emerged in 2014 to promote innovative techniques such as design-based thinking to approach policy problems in a new way. The aim was to involve a broader range of inputs and experts, where experimentation is the starting point to solving problems and developing options by trialling, testing and iterating constantly, with implementation in mind.^12^

In June 2017, the Alliance for a Cavity-Free Future (ACFF) along with the Dental Innovation and Translation Centre (DITC) at King's College London Dental Institute and the Policy Institute at King's held the first 24-hour long Dental Policy Lab (DPL1) to answer the question: 'How do we accelerate a policy shift towards increased resource allocation for caries prevention and control?'^13^

A problem with reorienting healthcare systems and persuading policymakers of the need to do this is that there are many different stakeholders, often with competing interests, and the systems that must change are complex and often fragmented.

**Fig. 1:**
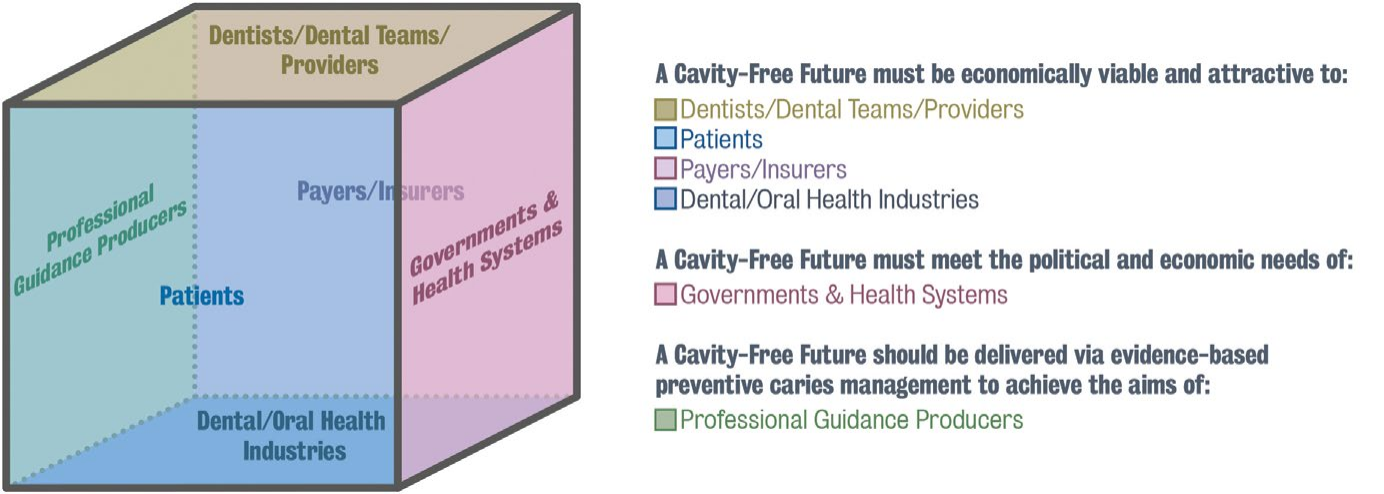
The Win-6 Stakeholder Cube, reproduced with permission from King's^13^

Through a series of iterative meetings, six main stakeholder groups were identified and 24 international experts from different backgrounds were invited to participate: dentists, government officials, public health specialists, professional guidance specialists, the oral health industry and health economists (Fig. 1).^13^ The resulting Lab took place over the course of two days. Several presentations and workshops including a range of participatory methods were held, to identify:

- The status quo and barriers to implementation
- The different 'patient types', based on their motivation and access to dental care
- The vision for a change and practical first steps that could be taken, tailored for each of the stakeholder groups
- The changes that would accelerate a shift of resources in favour of prevention for different patient types.

Participants were encouraged to think about each suggestion and discussion from the perspective of the six stakeholder groups. Once feedback was collated, the participants were then asked to propose concrete actions based on the findings. Following the Lab, a report outlining the outcomes was produced.^13^ The report was designed to be understood by all stakeholder groups including non-dental professionals.

## Results

After debate and discussions, the participants identified four areas in which immediate actions could be taken.

### 'Create prevention-based payment systems'

As most health systems are based on a fee-for-service structure, dental teams do not typically get paid to perform prevention. As payment systems have been shown to influence the practitioners' delivery of care,^11^^,^^14^ new models must be found to reward dentists for keeping their patients in a healthy state which also satisfy those managing dental healthcare systems.

Other types of payment systems have been described in the literature, such as capitation-based payment systems and pay-for-performance-based systems.^11^ There are several pros and cons for each, meaning that blending several system types may be an ideal solution to obtain better health outcomes for the patients, while maintaining access to dental care and respecting the financial stability of dental practices.^15^

Experiments such as the prototypes for new NHS dental contracts in England and France are examples of reforms that other countries can learn from, although some of the learning may be country-specific. Given the complexity of this outcome, a further Dental Policy Lab concentrating on this specific issue was held in 2018.^10^

### 'Expand and equip the dental workforce and increase interprofessional collaboration'

The existing workforce largely already knows the steps required to deliver effective preventive care, but this does not always happen in practice. This is partly to do with the failure of communication of recently updated knowledge. The participants agreed that work should commence immediately on strengthening messages and better utilising available tools to communicate effectively to ensure that dental teams are up to date on the most recent developments in preventive practice. Initiatives such as the International Caries Classification and Management System (ICCMS),^4^ a free-to-use, practice-based caries control and management system, and the subsequently developed CariesCare International, developed post-DPL1 in response to the call for improved, practice-friendly messaging, provide dental teams with actionable up-to-date evidence. In countries with well-established dental health systems, there are also initiatives looking to pilot closer cooperation between medical and dental teams.^16^^,^^17^

In some health systems, this may involve utilising other health workers and other professionals with access to patients, such as teachers or social workers, which would offer a wider base for the delivery of initial, preventive caries advice and care. An example of this would be training, mobilising and supporting health workers who usually work in other health domains to incorporate oral health assessment and onward referral into their routine contact with patients and the public.

### 'Shift public and industry behaviours'

Governments and policymakers must play a key role in influencing a change in public attitudes and behaviours towards sugar. The successful tactics employed with tobacco (for example, sugar taxation, advertising regulations, bans of sales in public places) might be used as a positive starting point. Evidence-based reviews on the effectiveness of the measures have been described,^18^ and may be used in different country settings. Additionally, the use of direct incentives to parents (such as giving bonus points to reduce insurance premiums) is already being successfully used in some countries.^19^

Different parts of the industry will also each have their role to play in terms of promoting healthy behaviours, products and developing the use of technology. The oral health industry and the dental products industry, as well as others, are all key to seeing improvement in health. This issue was also addressed further at the third Dental Policy Lab, held in 2019, titled 'Towards oral and dental health through partnership: how can the oral health and dental industries benefit from enabling positive behaviour in caries prevention and control among patients and the public?'^20^

### 'Demonstrate the value of a cavity-free world'

Investment in prevention is possible, but to demonstrate how much patients value this cavity-free health state, which would help policymakers understand the importance of oral health in terms of general quality of life, further studies are needed.

Though many professionals argue that investing in preventive care will save money, the cost of achieving a cavity-free future and what value it has for societies has not yet been described in the economic literature. While many dental and other professionals suspect that investing in prevention will prove worthwhile, economists argue that although preventive care may bring health benefits, it might not be cost-saving, as any reduction in spending due to reduced treatment may then be diverted to be spent on other treatments which produce less health benefit than those displaced. Even where cost savings could be genuine, there are still difficult decisions needed to find the resources needed for an initial investment in prevention, before cost savings are realised in the longer term.^21^ Rather than focusing simply on cost, arguments around the value placed by patients on better care (prevention, control and maintenance of healthy teeth) might be more persuasive.

Traditional methods that health economists rely on of quantifying the amount of health gain resulting from a measure are not useful for oral health. Demonstrating to policymakers, professionals and the public that a shift towards preventive care can, in the long term, be valuable and cost-effective both for the patient and the health system needs systematic economic and comprehensive clinical data that has not yet been collected. The DPL1 participants proposed to advocate setting up an 'economic competition' to collect and analyse reliable data on the value of patients being cavity-free.^13^

## Discussion

The Policy Lab is a novel collaborative technique to produce comprehensive policymaking, by gathering a broad range of stakeholders and facilitating interprofessional collaboration. These stakeholders, in the case of dentistry, are often in situations, such as dental contracts negotiations, where competing interests might keep important issues off the agenda or prevent forward movement. The working group allowed a facilitated discussion on a specific policy item: achieving a cavity-free future through resource shifts.

The creation of a comprehensive report, along with the inclusion of an overview infographic (Appendix 1) which summarises the Dental Policy Lab process and its key recommendations, has allowed effective communication with the breadth of stakeholders.

Although some of the actions proposed are targeted at the authorities, GDPs and the dental team have an essential role to play in several domains of actions:

- Contribute to the debate on contract reform. Talking with local policymakers about local solutions to increase prevention (for example, some regions in the UK have introduced specific/enhanced payments for prevention and initiated the process of contract reform). In France, stemming from the DPL1 discussions, dental Trade Unions began advocating for the reform of dental contracts and for a new payment system, and also for coverage by the National Health Insurance of fluoride varnishes for children
- Dental teams can orientate personal development plans and CPD to learning about prevention. Training is available in many organisations and resources can be found online^4^^,^^22^
- GDPs and their teams can liaise with local health visitors, GPs, paediatricians, schools, community groups, and ensure referral pathways to their practice for medical professionals who have concerns about oral health. Dental professionals should undertake an advocacy role, making local health decision-makers (such as in NHS England and local authorities) and those at a national level, through their MPs, aware of the need to encourage dental prevention through policies to shift public and industry behaviour
- Dental professionals should utilise arguments that go beyond simple cost-saving but also talk about the benefits of being cavity-free (for example, improved quality of life, improved productivity, better educational attainment and growth for children) when advocating for preventive approaches.

## Progress

Several positive initiatives developed since the DPL1 report was published in 2017, including the follow-up Dental Policy Labs and their outcomes, are reported on within this *British Dental Journal* Focus Issue. In addition to the further Labs, some of the impacts stemming from DPL1 include:

1. The FDI World Dental Federation Chief Dental Officers/Dental Public Health Section invited a report from the ACFF to its meeting during the 2017 FDI World Dental Congress in Madrid and has since embraced the ACFF as a partner, assisting in disseminating messaging and priorities to their networks
2. The British Dental Association considered the outcomes as a springboard to help guide their post-Minamata planning
3. The Office of the Chief Dental Officer England expressed an interest in incorporating some outputs into their 'Prototypes'
4. CariesCare International built their dental practice programmes around the recommendations of the report blueprint and have produced a 'CariesCare practice guide' with the *British Dental Journal* to advance the concepts recommended by the Policy Lab^23^
5. The Council of European Chief Dental Officers invited the ACFF to give presentations on the Dental Policy Lab to European Chief Dental Officers at their meeting in Cardiff in April 2018
6. An ACFF Health Economists Advisory Consortium/Dental Policy Lab Network was created, drawing together key thought leaders from Dental Policy Labs to continue the methodological and technical discussions around dental payment systems and future developments stemming from Policy Lab outcomes.

## Conclusions

Given the positive feedback and fast progress stemming from this initiative, the ACFF as well as other organisations found value in this multi-stakeholder approach. Bringing different perspectives together in this format proved to be an effective way to generate new ideas that can be translated into action. Stakeholders have continued the debate and are seeking to accelerate progress, for a significant opportunity to improve health and healthcare in dentistry. Second and third Dental Policy Labs were developed and actioned in subsequent years, with continued developments seen to date.

**Figure:**
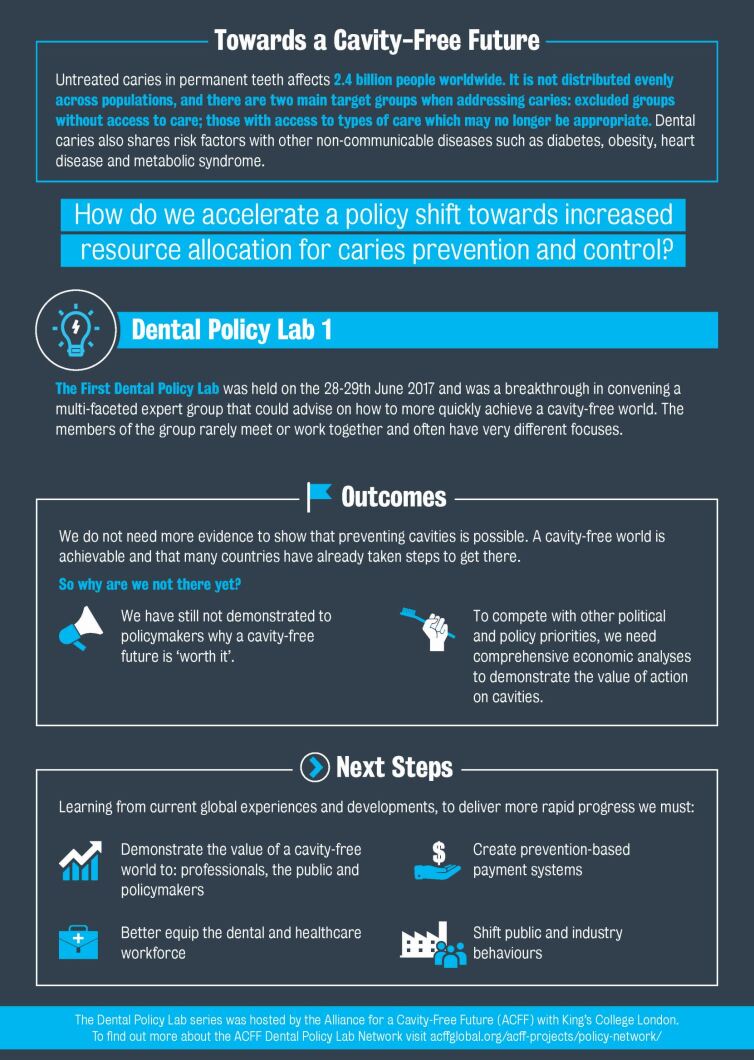
***Appendix 1*** Dental Policy Lab 1 overview infographic^13^

## References

[1] Kassebaum N J, Bernabe E, Dahiya M, Bhandari B, Murray C J, Marcenes W. Global burden of untreated caries: a systematic review and metaregression*.**J Dent Res* 2015; **94:** 650-658. 10.1177/0022034515573272 25740856

[2] Doherty R, Mike Curtis: It's criminal that we still have such high levels of childhood caries*.**Br Dent J *2018; **224:** 66-68. 10.1038/sj.bdj.2018.62 29372705

[3] Public Health England. *Research and Analysis-Hospital tooth extractions of 0 to 19 year olds*. London: Public Health England, 2019.

[4] Pitts N B, Ismail A I, Martignon S *et al.* ICCMS Guide for Practitioners and Educators. 2014. Available at https://www.iccms-web.com/uploads/asset/59284654c0a6f822230100.pdf (accessed June 2021).

[5] Doméjean S, Banerjee A, Featherstone J D B. Caries risk/susceptibility assessment: its value in minimum intervention oral healthcare*.**Br Dent J* 2017; **223:** 191-197. 10.1038/sj.bdj.2017.665 28798458

[6] Banerjee A, Frencken J E, Schwendicke F, Innes N P T. Contemporary operative caries management: consensus recommendations on minimally invasive caries removal*.**Br Dent J* 2017; **223:** 215-222. 10.1038/sj.bdj.2017.672 28798430

[7] Dawett B, Atkins B, Banerjee A. A guide to building 'MI' oral healthcare practice*.**Br Dent J* 2017; **223:** 223-227. 10.1038/sj.bdj.2017.673 28798433

[8] Birch S, Bridgman C, Brocklehurst P* et al.* Prevention in practice - a summary*.**BMC Oral Health* 2015; **15:** S12. 10.1186/1472-6831-15-S1-S12 PMC4580841 26391906

[9] Vernazza C R, Birch S, Pitts N B. Reorienting Oral Health Services to Prevention: Economic Perspectives*.**J Dent Res* 2021; **100:** 576-582. 10.1177/0022034520986794 PMC8138330 33478327

[10] Pitts N B, Mazevet M, Mayne C, Boulding H, Pow R. *Towards paying for health in dentistry: How can we create and implement acceptable prevention-based dental payment systems to achieve and maintain health outcomes?* London: King's College London, 2019.

[11] Brocklehurst P, Price J, Glenny A M* et al.* The effect of different methods of remuneration on the behaviour of primary care dentists*.**Cochrane Database Syst Rev* 2013; DOI: 10.1002/14651858.CD009853.pub2. 10.1002/14651858.CD009853.pub2 PMC6544809 24194456

[12] Policy Professional Board. *Twelve Actions to Professionalise Policy Making: A report by the Policy Profession Board.* London: HM Government, 2013.

[13] Pitts N B, Mazevet M E, Mayne C, Hinrichs S, Boulding H, Grant J. *Towards a Cavity Free Future: How do we accelerate a policy shift towards increased resource allocation for caries prevention and control? *London: The Policy Institute at King's, 2017.

[14] Malone A, Conway D I. Payment methods may influence behaviour of primary care dentists*.**Evid Based Dent* 2015; **16:** 4-5. 10.1038/sj.ebd.6401071 25909927

[15] Manski R, Moeller J, Chen H, Widström E, Listl S. Disparity in dental out-of-pocket payments among older adult populations: a comparative analysis across selected European countries and the USA*.**Int Dent J* 2017; **67:** 157-171. 10.1111/idj.12284 PMC5560036 28213893

[16] Cohen L A. Expanding the physician's role in addressing the oral health of adults*.**Am J Public Health *2013; **103:** 408-412. 10.2105/AJPH.2012.300990 PMC3673507 23327256

[17] Geriatric Medicine Research Collaborative. A nationwide survey of confidence and knowledge of assessment and management oral conditions among a sample of physicians, United Kingdom*.**BMC Res Notes* 2019; **12:** 348-348. 10.1186/s13104-019-4359-0 PMC6585010 31221209

[18] Public Health England. *Sugar Reduction The evidence for action*. London: Public Health England, 2015.

[19] Schmidt H, Gerber A, Stock S. What can we learn from German health incentive schemes? *BMJ* 2009; DOI: 10.1136/bmj.b3504. 10.1136/bmj.b3504 19778973

[20] Pitts N B, Pow R. *Towards Oral and Dental Health through Partnership: How can the oral health and dental industries benefit from enabling positive behaviour in caries prevention and control among patients and the public?* London: King's College London, 2020.

[21] Lord J, Longworth L, Singh J *et al*. Oral Health Guidance - Economic analysis of oral health promotion approaches for dental teams*.* 2015. Available at https://www.nice.org.uk/guidance/ng30/documents/oral-health-promotion-approaches-for-dental-teams-health-economic-analysis2 (accessed June 2021).

[22] ICCMS. ICCMS Caries Management. 2020. Available at https://www.iccms-web.com (accessed May 2021).

[23] Martignon S, Pitts N B, Goffin G* et al.* CariesCare practice guide: consensus on evidence into practice*.**Br Dent J* 2019; **227:** 353-362. 10.1038/s41415-019-0678-8 31520031

